# Improving the Classification of Alzheimer’s Disease Using Hybrid Gene Selection Pipeline and Deep Learning

**DOI:** 10.3389/fgene.2021.784814

**Published:** 2021-11-12

**Authors:** Nivedhitha Mahendran, P. M. Durai Raj Vincent, Kathiravan Srinivasan, Chuan-Yu Chang

**Affiliations:** ^1^ School of Information Technology and Engineering, Vellore Institute of Technology, Vellore, India; ^2^ School of Computer Science and Engineering, Vellore Institute of Technology, Vellore, India; ^3^ Department of Computer Science and Information Engineering, National Yunlin University of Science and Technology, Yunlin, Taiwan

**Keywords:** deep learning, Alzheimer’s disease, gene selection, gene expression, molecular bio-markers

## Abstract

Alzheimer’s is a progressive, irreversible, neurodegenerative brain disease. Even with prominent symptoms, it takes years to notice, decode, and reveal Alzheimer’s. However, advancements in technologies, such as imaging techniques, help in early diagnosis. Still, sometimes the results are inaccurate, which delays the treatment. Thus, the research in recent times focused on identifying the molecular biomarkers that differentiate the genotype and phenotype characteristics. However, the gene expression dataset’s generated features are huge, 1,000 or even more than 10,000. To overcome such a curse of dimensionality, feature selection techniques are introduced. We designed a gene selection pipeline combining a filter, wrapper, and unsupervised method to select the relevant genes. We combined the minimum Redundancy and maximum Relevance (mRmR), Wrapper-based Particle Swarm Optimization (WPSO), and Auto encoder to select the relevant features. We used the GSE5281 Alzheimer’s dataset from the Gene Expression Omnibus We implemented an Improved Deep Belief Network (IDBN) with simple stopping criteria after choosing the relevant genes. We used a Bayesian Optimization technique to tune the hyperparameters in the Improved Deep Belief Network. The tabulated results show that the proposed pipeline shows promising results.

## Introduction

Dementia is a broad term for a group of disorders with abnormal changes in the brain. The common forms of dementia interrupt the communication between the brain cells (Salat et al., 2001). When the communication between the cells is disrupted, the cognitive abilities, such as memory loss, feelings, thinking, and problem solving, behavior, and language proficiency of the individual will also be disrupted (Jo et al., 2019). Some of the common types of dementia are Parkinson’s disease, Lewy body dementia, Alzheimer’s disease (AD), Down’s syndrome, vascular dementia, dementia caused by alcohol, and HIV. Among these, 60–70% is accounted for by AD (Lawrence and Sahakian, 1995). Recently, there are increasing researches in the field of gerontology, a study of the physical aspects of aging. One such neurological disorder that appears in the elderly is the AD. Our work in this paper focuses on AD. AD is known to humankind for more than 100 years, yet the molecular mechanism and pathogenesis is far from fully understood (Reitz et al., 2011).

As commonly said, AD is a progressive, neurodegenerative brain disease, which is irreversible. The term progressive concerning AD means it gets worse over time because of irreversible degeneration of neurons (neurodegenerative) (Nussbaum and Ellis, 2003). In other words, the pathological change of AD is a slowly accumulating process. AD affects the hippocampus and cortex regions of the brain primarily. The primary reason for developing AD symptoms is more than the required accumulation of proteins around the brain cells (Wenk, 2003). The high levels of proteins make the communication between brain cells tedious. The actual reason for the onset of AD is still uncertain. Yet, few hypotheses were framed over the years, such as the accumulation of Tau and amyloid proteins, cholinergic, and genetics (Citron, 2010). Even with dominant symptoms, the dysfunctions of AD take years to be noticed, decoded, and revealed.

The early diagnosis starts with recognizing the mild cognitive impairment (MCI), which has a high possibility of causing AD (Liu et al., 2014). The onset of AD is commonly found around 65 years; however, early onset at a younger age is rare. Even after thorough research, the cause and progression seem to be uncertain (Huber et al., 2018). The proper diagnosis can be made only after the autopsy, yet, with advanced technologies in clinical screening, such as cerebrospinal fluid analysis, imaging techniques have led to early AD diagnosis. These methods provide inaccurate results, which delays the treatment at times (Wang and Liu, 2019). The limitations in clinical screening have led to the molecular data-based analysis. Identifying molecular biomarkers offers promising results, as it establishes accurate relationships between the phenotype and genotype symptoms. The accurate and early diagnosis of AD will help patients have the awareness and indulge in preventive measures, for instance, medications and changes in their lifestyle.

Although the molecular biomarkers offer better results than the clinical screening, the environmental and genetic factors should be taken into account. There are more than 1,000 even 10,000 features generated through transcripts, genes, proteins, and their interaction with each other (Moradifard et al., 2018). It is a considerable challenge to find the AD causing biomarkers from such Big data. Thus, machine learning and Artificial intelligence-based methods are focused on these days to meet the challenges. There is another issue with the molecular biomarkers; more than the volume, the dimensionality of the dataset increases faster (Tanveer et al., 2020). Molecular data, such as gene expression, are ultra-high dimensional datasets. The dimensionality is achieving higher levels of thousands and hundreds of thousands.

Meanwhile, the sample size did not witness the same amount of growth. Such a situation is commonly known as the High Dimensionality Low Sample Size (HDLSS) problem or “curse of dimensionality”. The machine learning techniques widely used are not suited for such cursed dimensional data (Lee and Lee, 2020). The inconsistent ratio between the number of features and the number of samples will lead to overfitting, incompatible algorithm, and extended computational time.

To solve the curse of dimensionality problem, feature selection is proposed as a solution. In this study, we develop a gene selection pipeline combining filter, wrapper, and unsupervised method to select the relevant features in causing AD. Later, the selected genes are passed through the Improved Deep Belief Network (IDBN), which is implemented to classify the AD and non-AD individuals. The selection of relevant features will make the classification of AD and non-AD individuals accurate and easy.

## Background and Motivation

### Alzheimer’s Disease and Machine Learning Algorithms

The most widely used technique in diagnosing AD is the clinical screening methods, such as brain imaging. At times, the clinical screening methods provide inaccurate results due to technical errors, which eventually delays the treatment. Hence, the research is gradually moving towards molecular data, for instance, microarray data. In the process of finding out differentially expressed genes, thousands of genes are captured and monitored to evaluate the effects of a disease or a treatment (Fung and Stoeckel, 2007). For detecting the expression of hundreds and thousands of genes simultaneously, microarray technology is used widely. In microarray, thousands of genes or DNA sequences are printed in already defined positions. The DNA microarray datasets have vast volumes of genes captured, which might not be relevant to the undertaken domain (treatment/disease) (Huang et al., 2018).

Considering the huge volume of features involved, machine learning-based methods help greatly in classifying AD patients from healthy individuals. Machine learning is a continuously growing area of research, advantageous in many domains, mainly in healthcare. Machine learning algorithms are trained on a set of data, learn from the data, find out the patterns, and predict the future possibilities without much human intervention (Orimaye et al., 2017). It is a part of Artificial Intelligence, assists in data analysis, and automates model building. There are three categories of machine learning algorithm based on the dataset used (Hutter et al., 2019): supervised learning, when the data are structured and attributes are labeled; unsupervised learning, when the data are unstructured and the attributes are unlabeled; and semi-supervised/semi-unsupervised learning, when the data are a combination of supervised and unsupervised categories. Although machine learning algorithms offer great assistance in finding patterns and classification, it is not suitable when the ratio of sample to feature is largely different. In that case, machine learning algorithms will have an overfitting problem.

### Related Works

Artificial Intelligent models have been widely deployed in genetics research (Mahendran et al., 2020). Deep learning approaches remove certain data pre-processing, which is usually deployed in machine learning (Srinivasan et al., 2017; Agarwal et al., 2018; Chakriswaran et al., 2019; Khan et al., 2021a; Khan et al., 2021b; Khan et al., 2021c) (Sanchez-Riera et al., 2018; Srinivasan et al., 2020; Afza et al., 2021; Ashwini et al., 2021; Attique Khan et al., 2021; Khan et al., 2021d; Mamdiwar et al., 2021; Srinivasan et al., 2021). AD is a neurological disorder identified through brain imaging, and there are many works focused on classifying AD through brain images with the help of machine learning or deep learning techniques. For instance, Convolutional Neural Network (CNN) and LeNet architecture is applied on the MRI data to classify AD (Sarraf and Tofighi, 2016a). There are many such works focused on classifying AD through the brain images (Sarraf and Tofighi, 2016b; Farooq et al., 2017; Ji et al., 2019; Ramzan et al., 2020; Tufail et al., 2020), though the imaging data provide inaccurate results at times. Thus, the focus recently is shifted to the molecular dataset such as the Gene Expression and DNA Methylation data, though the problem with such data is the dimensionality. There is a huge number of features, yet very small sample size.

Therefore, the research is focused more on the gene selection techniques to select the relevant features in classifying the AD. For instance, Park et al. (Park et al., 2020) implemented machine learning-based gene selection and a deep learning classifier combining the gene expression and DNA Methylation datasets. Also, the gene pair interaction-based research is done to identify the biomarkers accurately to classify the AD (Chen et al., 2019). Furthermore, there are approaches implemented to detect the possible progression of a dementia to AD with the help of machine learning techniques (Martínez-Ballesteros et al., 2017; Miao et al., 2017). Also, the artificial intelligence approaches are adopted in precision medicine to validate the drugs for AD.

### Feature Selection

There are four mainly used feature selection techniques, Filter, Wrapper, Hybrid, and Ensemble (Bashir et al., 2019). Filter-based techniques are independent of the classifier model and computationally efficient at times (Acharya et al., 2019). The search for relevant features is isolated completely from the classifier model. The features with the lowest relevance score are eliminated. The filter methods are further classified into univariate and multivariate filters, where univariate treats and evaluates the features individually and multivariate evaluates the feature dependencies. The wrapper methods are implemented as a part of the classification model (Zhou et al., 2018). The feature subsets selected are validated through training and testing datasets. The features with maximum evaluation score are selected for the final classification. The wrapper method’s major drawbacks are as follows: it demands high computational time, it is classifier dependent, and overfitting (Mirzaei et al., 2018).

The ensemble methods simultaneously build different feature subsets and combine the results using standard aggregate methods, such as majority voting, sum rule, mean rule, and weighted voting (Pes, 2019). The exponential growth of technologies across all the domains created a data explosion, which is continuously spreading at an unprecedented speed. The previously mentioned feature selection methods are not suitably designed for a dataset with HDLSS problem and unstable and not robust with changing inputs. Thus, ensemble methods are designed aiming to bring more robustness and stability to the model (Neumann et al., 2017). The main goal of the ensemble model is to attain a better trade-off between stability and predictive performance. The ensemble methods are generally grouped under homogeneous and heterogeneous methods. The homogeneous algorithms use selection algorithm with the varying dataset, for instance, boosting or bagging. The homogeneous ensembles handle the stability issues better. The heterogeneous ensembles implement different selection algorithms with the same dataset. In both cases, the output will be combined to a single feature set, which probably provides an optimal solution (Pes, 2019). Apart from homogeneous and heterogeneous methods, there is another group called the hybrid, which uses different selection algorithms with other datasets.

Though these three feature selection methods are needed, there are various reasons that make them unreliable, unstable, and sometimes ignore the algorithms’ stability. However, there is a fourth method that is focused on much these days, the hybrid method. To solve the issues with respect to filter and wrapper methods, a hybrid method is introduced. It combines two or more feature selection techniques and produces a new method with added benefits. In most cases, wrapper and filter methods are made hybrid by combining their advantages (Hoque et al., 2018; Kollias et al., 2018; Thavavel and Karthiyayini, 2018). This study implemented a feature selection pipeline for selecting relevant genes from the raw Alzheimer’s gene expression dataset. The filter method is simple and ignores the feature dependencies most of the time and also occasionally includes the redundant features. Wrapper methods are at high risk of overfitting and are stuck in the local optima. It is also computationally intensive. Ensemble methods are better than filter and wrapper; however, on the dataset with the High Dimensional and Low Sample Size (HDLSS) issue, it does not perform well. Thus, we desired to implement a feature selection method catering to the HDLSS issue. Hybrid methods are flexible and robust upon high-dimensional data. Also, they offer higher performance and better computational complexity than the filter and wrapper methods. The pipeline consists of a filter method, wrapper method, and unsupervised gene selection method.

## Dataset and Resources

For a better treatment of AD, the gene expressions are captured preferentially during normal neurological aging (Lima et al., 2016; Carpenter and Huang, 2018). The data captured during the course of AD will improve the understanding of the underlying pathogenesis of AD. This practice will help in the early diagnosis and treatment of AD. The dataset (GSE5281) (Liang et al., 2007) used in this study is from one of the widely accessed data repository, Gene Expression Omnibus (GEO). The dataset consists of information about AD and normal aged brain with 161 samples and 54,675 features (gene expression). The gene expressions are captured from six brain regions of *Homo sapiens* using the LCM cells on the Affymetrix U133 plus 2.0 array with approximately 55,000 transcripts. Among the 161 records, 74 controls and 87 are affected. We have used RStudio for implementing the mentioned approaches in this study. To analyze the gene expression dataset, R has many beneficial packages such as the Bio-conductor.

## Methodology

**FIGURE 1:**
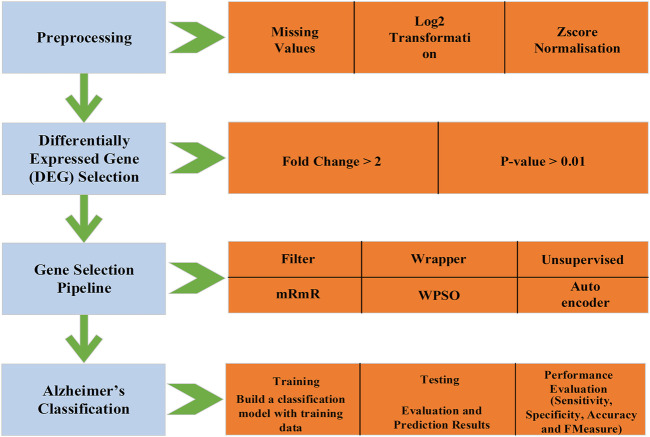
Process flow diagram—proposed system.

We implemented a gene selection pipeline by combining a filter (mRmR), wrapper (Wrapper-based PSO), and unsupervised method (Autoencoder). The mRmR eliminates the genes with maximum redundancy (high correlation among themselves) and the selected genes are inputted to the Wrapper-based PSO, which has k-means as the wrapper method and selects the relevant genes. The selected genes are passed through an autoencoder for further compression. The compressed genes are used for classifying the AD and non-AD individuals using the IDBN. The process flow of the proposed framework is shown in Figure 1.

### Minimum Redundancy and Maximum Relevance (mRmR)

mRmR is the most widely used practical statistical approach for feature selection. It was proposed by Peng et al. (Ding and Peng, 2005) initially for classifying patterns. The mRmR method strives to choose the feature subset that is highly relevant to the outcome (target class) and minimally redundant. In simple terms, the features are highly similar to the outcome class (relevance) and dissimilar among themselves (redundancy). The feature selection process in mRmR is carried out by adding the features with the highest feature importance to the feature list at each step (El Akadi et al., 2011; Billah and Waheed, 2020).

The aim of mRmR in gene selection is to select a gene subset, G_s_, with {X_m_} features that are highly correlated with the target class T (output class). The mRmR involves three steps, finding the relevance, finding the redundancy, and combining the two to get the mRmR feature set.

Step 1 Maximum Relevance

The maximum relevance is calculated using the mean of Mutual Information of all the features in Xm with target class T. The Mutual Information between R and S random variables can be calculated by

$$MI\left(R,S\right)=\;\sum_{r\in ΩR}\sum_{s\in ΩS}p\left(r,s\right)log\frac{p\left(r,s\right)}{p\left(r\right)p\left(s\right)}$$ (1)

Where.

R—response variable.

S—number of features.

ΩR and ΩS—sample spaces with respect to R and S,

p (r, s)—joint probability density, and

p ()—marginal density function.

The maximum relevance is given by

$$Relevance\left(G_{s}\right)=\;\frac{1}{\left|G_{s}\right|}\sum_{X_{m}\in G_{s}}MI\left(T,X_{m}\right)$$ (2)

Where MI(T, X_m_)—Mutual Information of feature X_m_ with class T.

Step 2 Minimum Redundancy

The minimum redundancy is calculated with the formula

$$Redundancy\left(G_{s}\right)=\frac{1}{{\left|G_{s}\right|}^{2}}\sum_{X_{i},X_{j}\in G_{s}}MI\left(X_{i},X_{j}\right)$$ (3)

Step 3 Combining the above two constraints

The maximum relevance and minimum redundancy are combined to form the mRmR using the formula

$$\max\alpha \left[\mathrm{Relevance}\left(G_{s}\right),\mathrm{Redundancy}\left(G_{s}\right)\right]$$ (4)

Where α = (Relevance (G_s_) − Redundancy (G_s_))

### Particle Swarm Optimization (PSO)

PSO is a stochastic, metaheuristic algorithm inspired by the birds’ swarming behavior. From the birds’ flocking behavior, it is understood that each individual is affected by the leader or the global optima and the personal performance or the local optima (Deepthi and Thampi, 2015). The PSO is an optimization technique based on population proposed by Eberhart and Kennedy (Kennedy and Eberhart, 1995), successfully applied in many global search problems. It is considered in many feature selection problems as it is easy to implement, and has reasonable computational time, global search, and fewer parameters.

In PSO, the population is initialized with particles, each having its own position and velocity. The quality of the particles is estimated at each iteration with the help of a fitness function. Every particle in the search space will carry the present position (x_ppos_), present velocity (v_pvel_), and personal best (y_pbest_). After every iteration, the velocity can be updated by

$$v_{pvel}\left(new\right)=i_{w}v_{pvel}\left(old\right)+a_{c1}r_{1}\left(y_{pbest}-x_{ppos}\left(old\right)\right)+a_{c2}r_{2}\left({\hat{y}}_{gbest}-x_{ppos}\left(old\right)\right)$$ (5)

Where.

i_w_—inertia weight,

a_c1_ and a_c2_—acceleration constants,

r_1_ and r_2_—random numbers (range [0, 1]),

v_pvel_ (old)—present best solution of the particle,

y_pbest_—personal best solution of the particle, and

ŷ_gbest_—global best solution.

The new position of the particle can be determined by

$$x_{ppos}\left(new\right)=x_{ppos}+v_{pvel}\left(new\right)$$ (6)

The positions and the velocity of the particle are updated at every iteration using the formulas given. The process is stopped when certain minimized fitness function criteria are achieved or a particular predefined iteration is reached. For position and velocity updates, the particles use the knowledge of their own and that of other neighboring particles. The final output represents the optimal feature set. We implemented a wrapper-based PSO with the k-means algorithm as the wrapper method. This wrapper method will aid in overcoming the problem of reaching local optima.

The fitness function for each subset is calculated using the below equation,

$$Sum\;of\;Squared\;Error=\sum_{x=1}^{k}\sum_{i\in C_{x}}d^{2}\left(c_{x},i\right)$$ (7)

Where.

k—number of clusters,

i—object in the cluster,

c_x_—cluster centroid, and

d—Euclidean distance.

### Autoencoders

Autoencoder is an artificial neural network with feed-forward processing. The autoencoder consists of input and output with one or more hidden layers, where the number of neurons (features) in the input and output layer is the same (Chicco et al., 2014). The autoencoder’s main aim is to reconstruct the inputs such that the difference between the input and the output is minimized. The learning in autoencoder is compressed and distributed (encoding) (Ferri et al., 2021). The training of autoencoder involves three steps:

1. If “x” is the input and “x̂" is the output, the feed-forward pass is done to estimate the values of all the nodes in the hidden layers after applying the activation function. For an autoencoder with a single hidden layer, the hidden unit vector h_u_ is given by

$$h_{u}=a_{func}\left(W_{e}.x+bias_{e}\right)$$ (8)

Where.

h_u_—hidden unit,

a_func_—activation function.

W_e_—parameter matrix (encoding),

x—input, and

bias_e_—bias parameter vector (encoding).

2. Map the hidden representation into the space “x” with the help of the decoding function. The decoding function is given by

$$\hat{x}=a_{func}\left(W_{d}.h_{u},\;+bias_{d}\right)$$ (9)

Where.

W_d_—parameter matrix (decoding) and

bias_d_—bias parameter vector (decoding).

3. Calculate back propagation error using the formula

$$MSE\left(x,\hat{x}\right)={\left\|x-\hat{x}\right\|}_{2}^{2}={\left\|x-\left(W_{d}.h_{u}+bias_{d}\right)\right\|}_{2}^{2}$$ (10)

### Deep Belief Network

In Deep Belief Network (DBN), the Restricted Boltzmann Machines are stacked together to form a network (An et al., 2020). RBMs are energy-based generative models with two layers, visible and hidden. Both the layers have nodes connected to each other (Mahendran et al., 2020; Sureshkumar et al., 2020). The major components in RBMs are bias, weight, and activation function (Le Roux and Bengio, 2008; Sekaran and Sudha, 2020). The output is produced after processing with the activation function. We implemented an IDBN with stopping criteria. We chose the hyperparameters using the Bayesian Optimization technique. The Bayesian approach for tuning the hyperparameter keeps past records and verifies the probability to select the next set of parameters. It takes informative decisions in choosing the parameters. The final values for the hyperparameters in IDBN are as follows: learning rate = 0.01, hidden layers = 9, number of nodes per layer = 342, and dropout rate = 0.85. We used the Rectified Linear Unit (RLu) as the activation function. To avoid the overfitting problem, we introduced a stopping criteria strategy. After every 40 epochs, the test accuracy of the last 10 epochs will be compared and checked for convergence, and the training accuracy will also be checked. If both the conditions are satisfied, the learning is ended.

### Evaluation Metrics

For evaluating the results of the proposed model, we have used the standard evaluation metrics such as Accuracy, Sensitivity, Specificity, and FMeasure.

• Accuracy: It is a simple ratio between the correctly classified as AD and non-AD to the total number of samples.
• Sensitivity: It is a measure to identify correctly those with AD.
• Specificity: It is a measure to identify correctly those without AD.
• FMeasure: It is the weighted average of recall and precision (the percentage of samples that are classified as AD positive and are actually positive).

#### Pseudocode

mRmR—WPSO—AE

mRmR.

Input: Candidates (set of initial genes).

Step 1: for genes *g*_*i*_ in candidates do.

Step 2: relevance = calculate the relevance score using Eq. 2;

Step 3: redundancy = 0;

Step 4: for genes *g*_*j*_ in candidates do.

Step 5: redundancy = calculate the redundancy score using Eq. 3;

Step 6: end for.

Step 7: *mrmr_values* [*g*_*i*_] = Eq. 4;

Step 8: end for.

Step 9: selected_genes = take (number_of_genes_required);

WPSO.

Step 10: Initialize *x* random gene subsets from the selected_genes with *y* number of genes in each subset.

Step 11: For every random subset *x*, initialize position and velocity vectors.

Step 12: Cluster initial subset with K = *k* using k-means clustering.

Step 13: Evaluate the fitness_value using Eq. 7

Step 14: Based on the fitness function, update the subset’s *pbest* and *pbestloc*.

Step 15: repeat.

Step 16: if (*fitness_value < pbest*) then.

Step 17: update *pbest* and *pbestloc*;

Step 18: end if.

Step 19: Initialize *gbest* and *gbestloc* after finding the minimum *fitness_value* in all the subsets.

Step 20: for j = 0 to *swarm_size-1* do.

Step 21: Estimate velocity using Eq. 5;

Step 22: Update subset location using Eq. 6;

Step 23: end for.

Step 24: Set the *fitness_value* by computing the squared error using the present location of the gene subset.

Step 25: until predefined number of iterations reached;

Output: Best subset of genes (*gbestloc*).

Autoencoder.

Input: *gbestloc_matrix* (*GM*) ∈ {0, 1}^m×n^, where m and n are genes and features.

Step 26: Initialize hidden units (h_u_), where h_u_ < *m*, and hidden layers (d).

Step 27: Training:

Step 28: for each GM_i_ (gene profile) of GM, where i ∈ [1, m].

Step 29: for each hidden layer d.

Step 30: compute hidden activation function using Eq. 8

Step 31: reconstruct the output using Eq. 9

Step 32: evaluate the error using Eq. 10

Step 33: update the weight by back propagating the error.

Step 34: Testing:

Step 35: for each GM_i_ (gene profile) of GM, where i ∈ [1, m].

Step 36: autoencode GM_i_ and produce. ${\hat{GM}}_{i}$

Step 37: set ${\hat{GM}}_{i}$ as *i*th row of the output matrix. $\tilde{GM}$

## Results and Discussion

**FIGURE 2:**
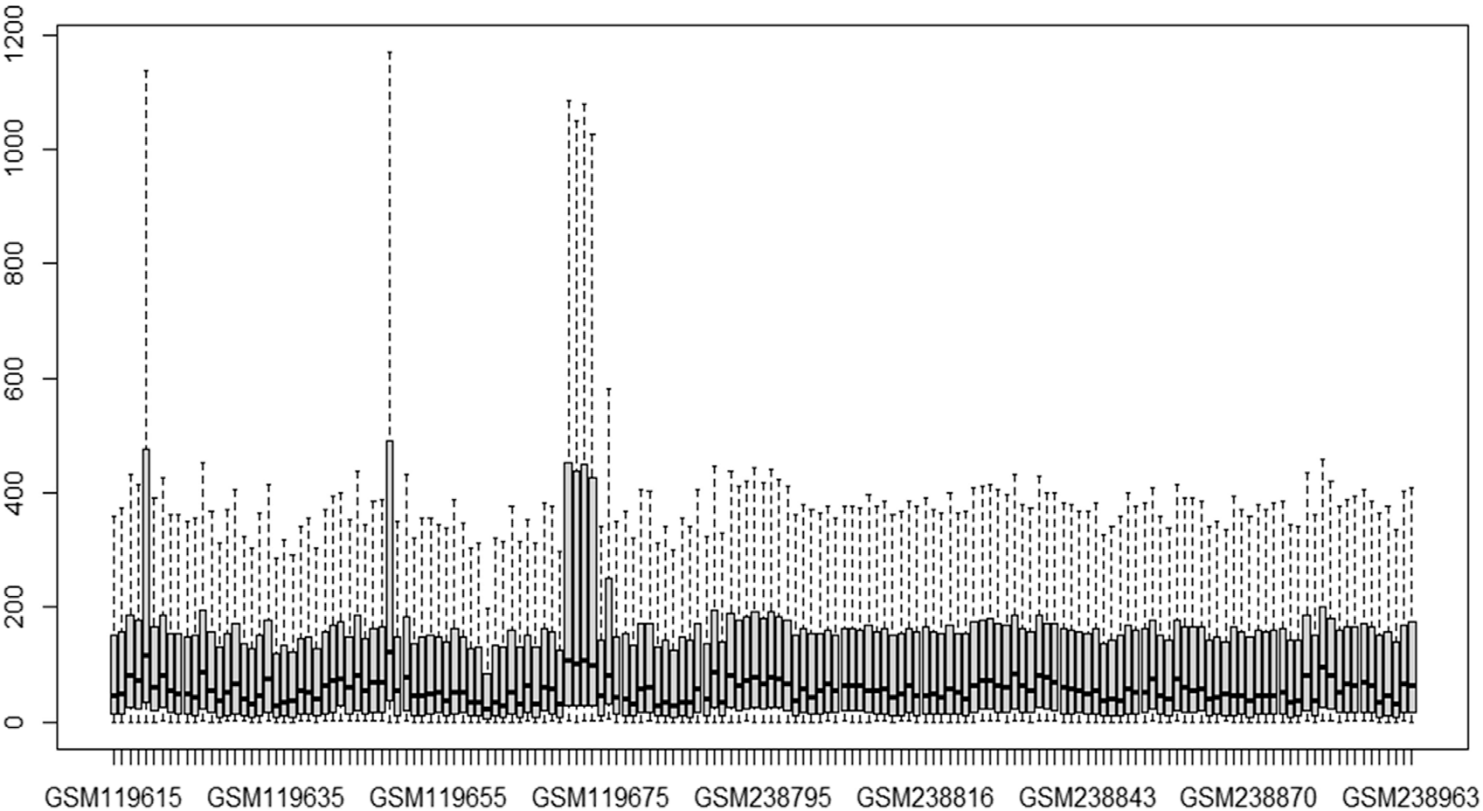
Boxplot of gene expression data before transformation.

**FIGURE 3:**
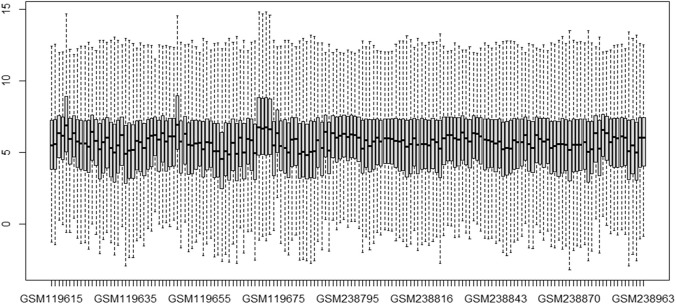
Boxplot of gene expression data after transformation.

**FIGURE 4:**
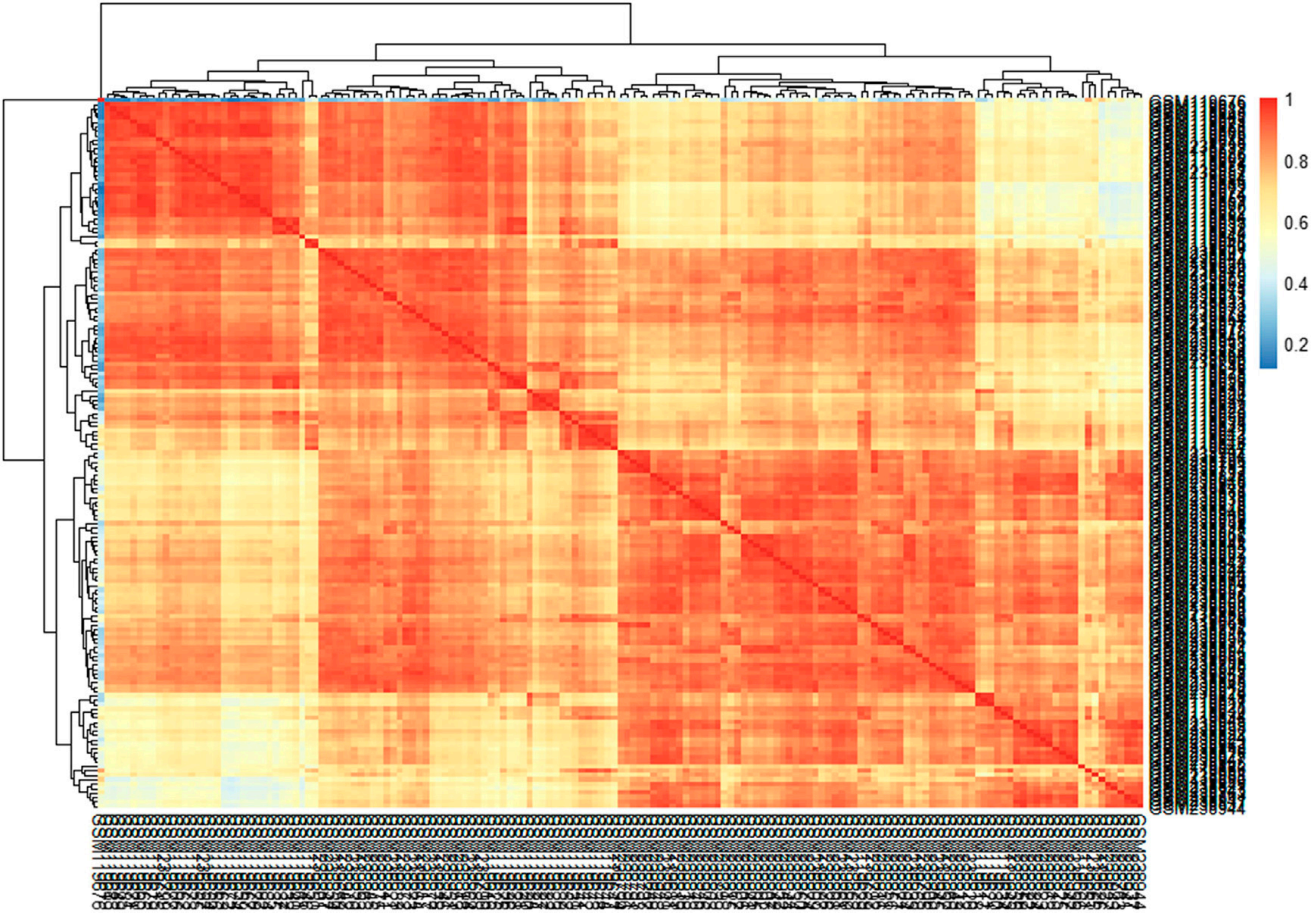
Heat map of differentially expressed genes.

**FIGURE 5:**
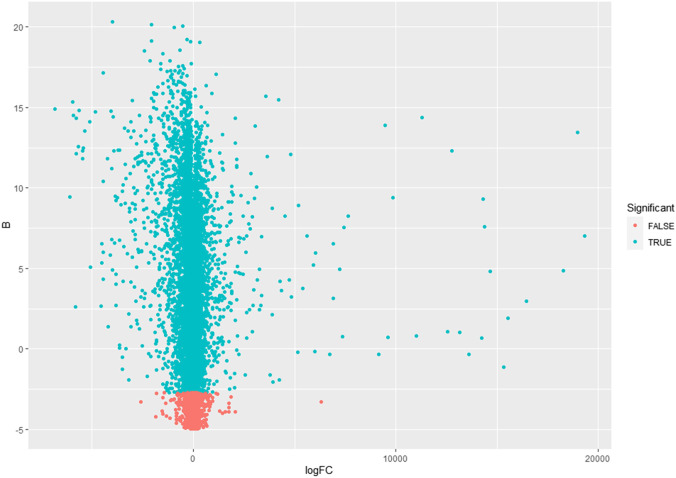
*p*-value and fold change plot.

This study’s primary aim is to improve the classification accuracy of the model in classifying Alzheimer patients by selecting the most relevant feature subset. The dataset used in this study has 161 samples and 54,675 features. The raw gene expression level data are highly skewed, as can be seen in the box plot shown in Figure 2. Thus, we applied log2 transformation to make it symmetrical. The results after applying the log2 transformation can be seen in the box plot shown in Figure 3. We applied *Z*-score normalization on the transformed data to make it comparable across all the platforms. Once the data are normalized, the differentially expressed genes are identified with fold change and *p*-value. The threshold used for fold change and *p*-value is |FC| > 2 and *p*-value > 0.01. The heat map from Figure 4 shows the levels of differentially expressed genes. The plot from Figure 5 shows the *p*-value and fold change levels of the differentially expressed genes. False represents the expression levels that are below the threshold, and true represents the expression levels that are above the threshold. The respective genes are selected and carried forward for the next stage, which is feature selection.

We designed a feature selection pipeline with mRmR, PSO, and autoencoder. The mRmR selects the gene with maximum relevance and minimum redundancy. Then, we applied a wrapper-based PSO technique with k-means clustering as the wrapper to further select the candidate genes. The candidate genes selected are passed through an autoencoder to form the latent representation of the provided input, compress the data without much information loss, and then rebuild as output with as low error as possible. The primary goal of passing the genes through autoencoder is to make the data less sensitive to variations in training. After selecting the relevant features (CTD-3092A11.2, CHGB, JPX, MAFF, AC004951.6, APLNR, MT1M, SST, PCYOX1L, PRO 1804, and SLC39A12), we implemented an IDBN.

We used the leave-one-out cross-validation method to evaluate the proposed model (Srinivasan et al., 2019). Leave-one-out validation is used because the sample size is less than the feature size. The metrics we used to evaluate the model are Sensitivity, Specificity, Accuracy, and FMeasure. We compared the results of the proposed feature selection pipeline with widely used Principal Component Analysis (PCA), Correlation-based Feature Selection (CBFS), and minimum Redundancy and maximum Relevance (mRmR). We also implemented two linear and two non-linear classifiers Support Vector Machine (SVM), Linear Discriminant Analysis (LDA), Naïve Bayes (NB), and Multi-Layer Perceptron (MLP), to compare the results with the IDBN.

**FIGURE 6:**
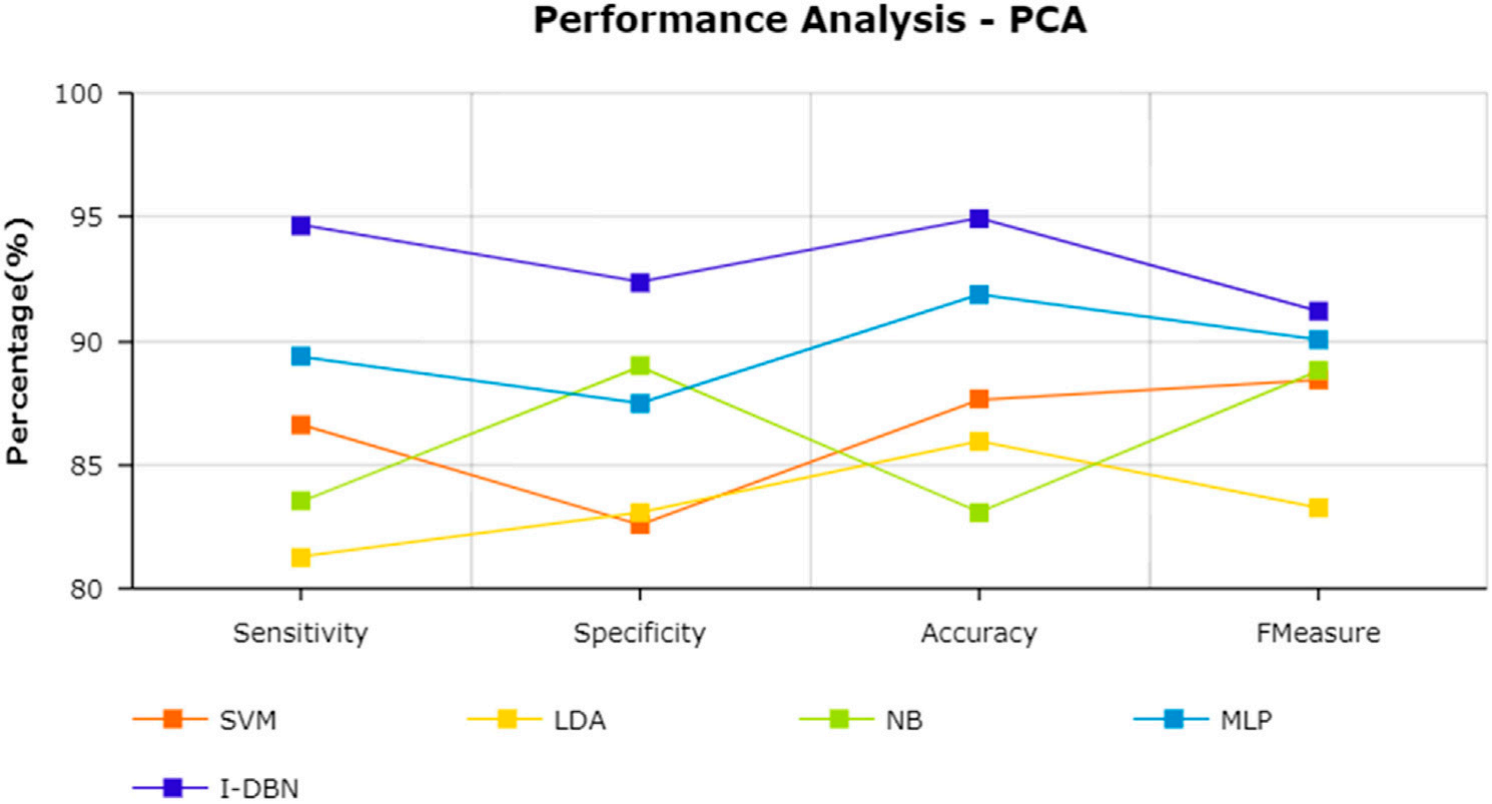
Performance analysis—PCA.

**FIGURE 7:**
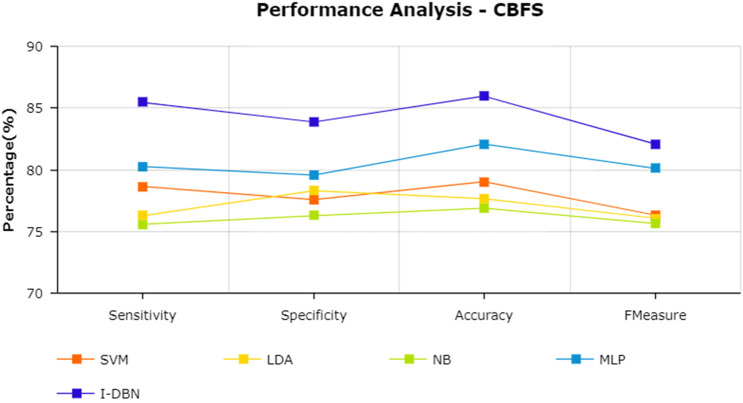
Performance analysis—CBFS.

**FIGURE 8:**
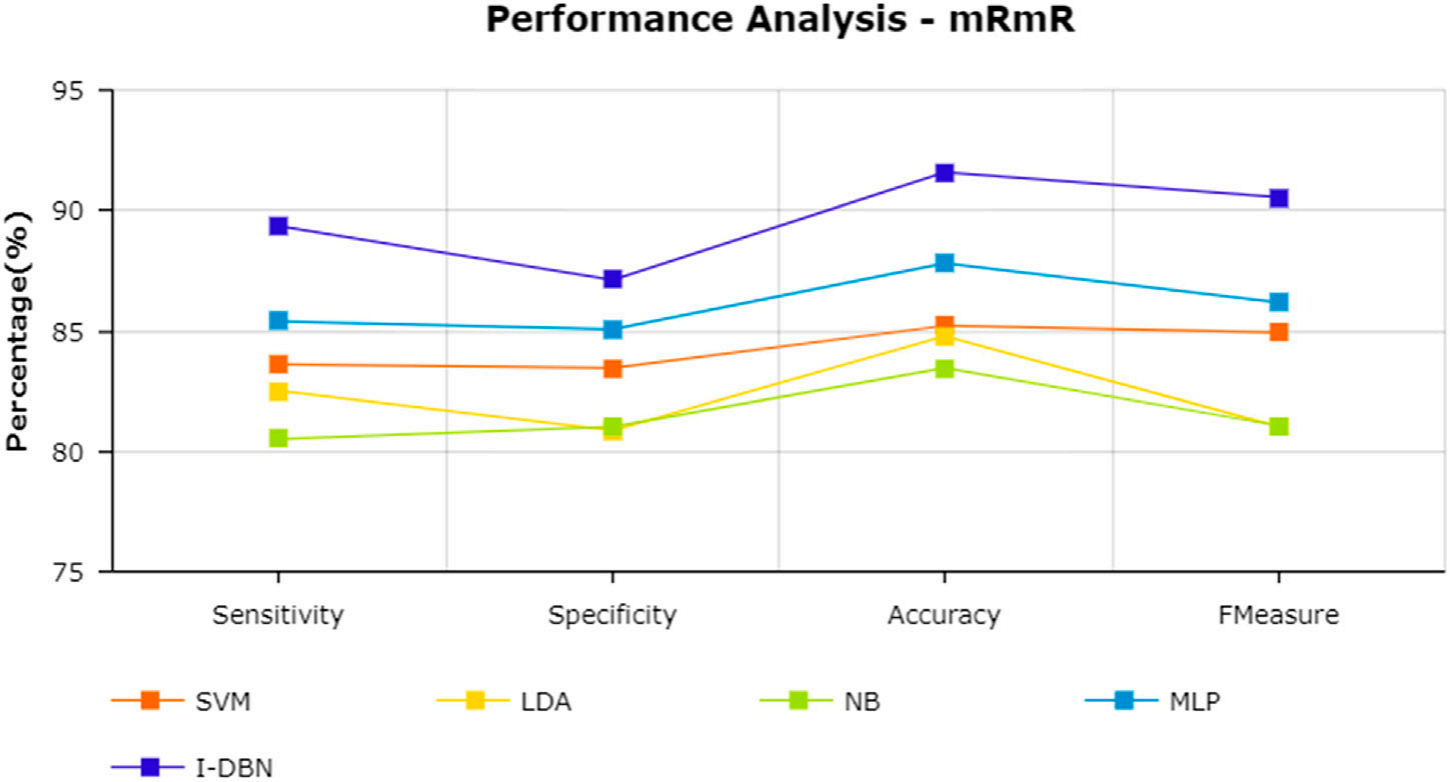
Performance analysis—mRmR.

**FIGURE 9:**
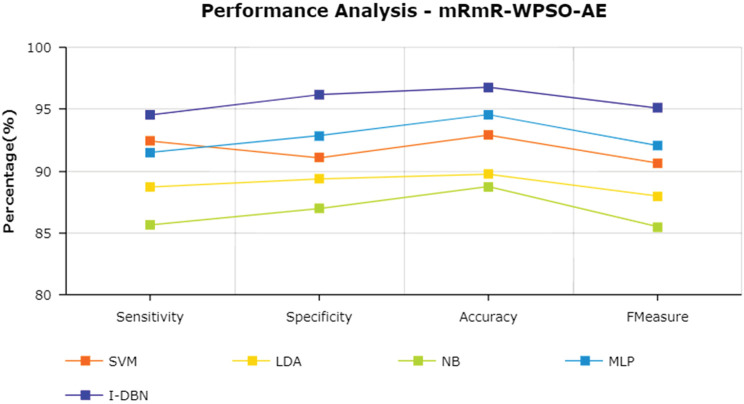
Performance analysis—mRmR-WPSO-AE.

**FIGURE 10:**
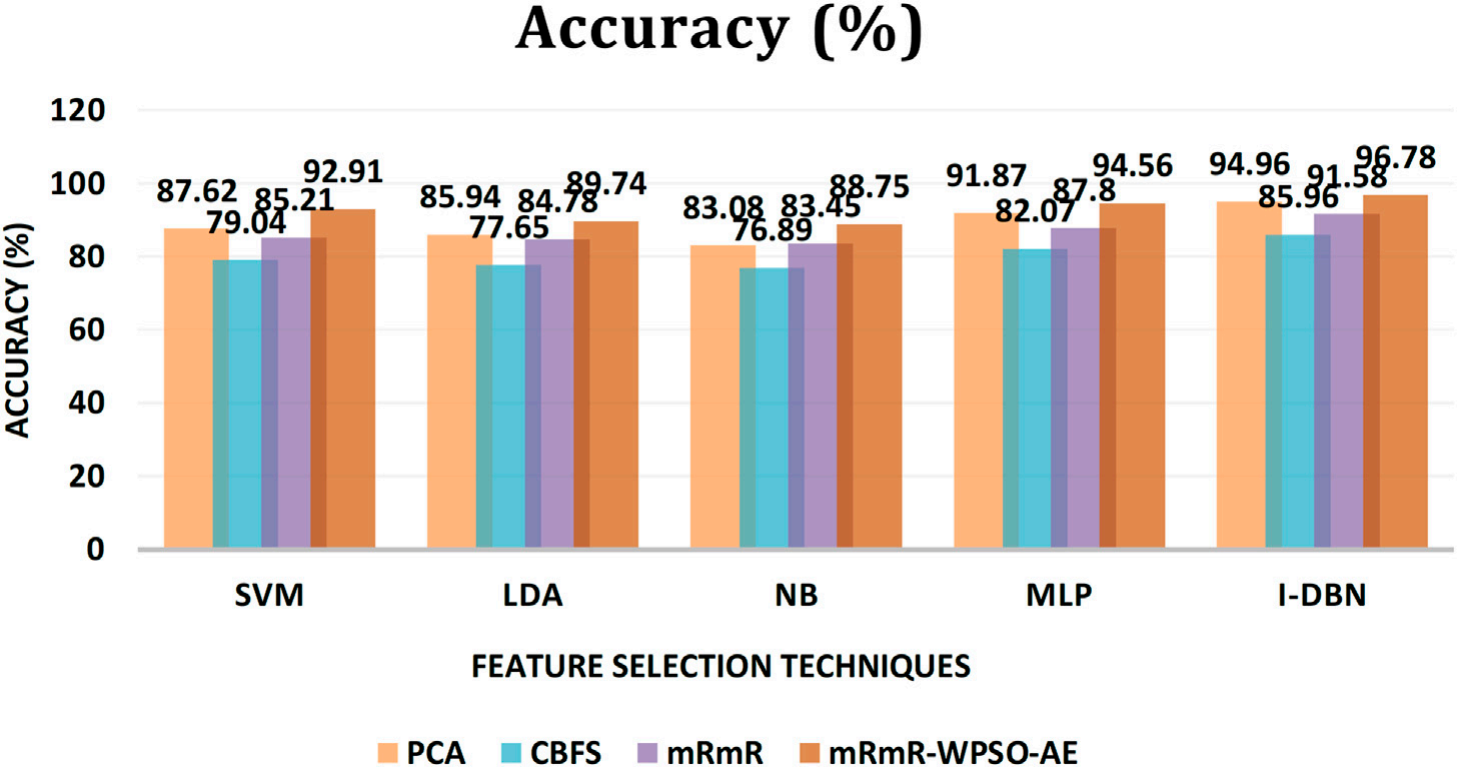
Accuracy comparison of the implemented models.

The results are tabulated in Table 1. The tabulated results show that the proposed feature selection algorithm pipeline (mRmR-WPSO-AE), along with IDBN, classifies Alzheimer’s slightly better than the other implemented models. The linear models SVM and LDA produce an accuracy of 92.91 and 89.74% with the proposed gene selection pipeline, which is better than the PCA’s 87.62% (SVM), 85.94% (LDA), CBFS’s 79.04% (SVM), 77.65% (LDA), and mRmR’s 85.21% (SVM), 84.78% (LDA). Also, with the non-linear models, NB and MLP produce an accuracy of 88.75 and 94.56%, which is again better than the PCA’s 83.08% (NB), 91.87% (MLP), CBFS’s 76.89% (NB), 82.07% (MLP), and mRmR’s 83.45% (NB), 87.80% (MLP). SVM performs better among the linear models than LDA along all the implemented gene selection methods, and MLP performs better than NB in the non-linear category. The combination of the proposed gene selection pipeline (mRmR-WPSO-AE) and IDBN shows the promising result with 96.78% accuracy in classifying Alzheimer’s patients. From the plots shown in Figures 6–9, it is clear that IDBN shows slightly better results than the other implemented classification models. The plot from Figure 10 shows the Accuracy comparison of the implemented models. The plot shows that the IDBN and mRmR-WPSO-AE have better accuracy than the other models.

**TABLE 1:** Results analysis.

Feature selection technique	Evaluation metrics	SVM	LDA	NB	MLP	IDBN
PCA	Sensitivity	86.61	81.28	83.50	89.35	94.68
	Specificity	82.57	83.07	88.96	87.48	92.38
	Accuracy	87.62	85.94	83.08	91.87	94.96
	FMeasure	88.42	83.26	88.78	90.05	91.20
CBFS	Sensitivity	78.65	76.27	75.58	80.26	85.46
	Specificity	77.57	78.31	76.27	79.56	83.87
	Accuracy	79.04	77.65	76.89	82.07	85.96
	FMeasure	76.32	76.07	75.63	80.10	82.08
mRmR	Sensitivity	83.61	82.51	80.51	85.40	89.36
	Specificity	83.45	80.87	81.02	85.07	87.12
	Accuracy	85.21	84.78	83.45	87.80	91.58
	FMeasure	84.93	81.02	81.07	86.18	90.55
mRmR-WPSO-AE	Sensitivity	92.43	88.71	85.64	91.51	94.54
	Specificity	91.07	89.36	86.97	92.87	96.17
	Accuracy	92.91	89.74	88.75	94.56	96.78
	FMeasure	90.64	87.98	85.50	92.06	95.09

## Conclusion and Future Work

Alzheimer’s is a progressive degenerative brain disease in the elderly. It is difficult to diagnose even with dominant symptoms. The proper diagnoses are made only during an autopsy after the death of the individual. Recent advances have made it easy to be detected early, using clinical screening with technologies such as brain imaging. Although brain imaging proves effective in most cases, in some cases, the results are inaccurate. The inaccuracies in the results make it difficult for early diagnoses and appropriate treatment for the individual. Thus, the research now shifts to molecular biomarker identification, which helps to differentiate clearly between genotype and phenotype characteristics.

The molecular data-based research proves to be effective. Still, it generates huge volumes of data consisting of transcripts, transcriptomes, etc. It creates a “curse of dimensionality” problem. Thus, machine learning-based feature selection techniques are implemented to select only the relevant genes affecting the target class (outcome). We implemented one such gene selection method for choosing the relevant genes. We designed a hybrid gene selection pipeline by combining mRmR, WPSO, and AE. We compared the results with other commonly used feature selection techniques, such as PCA, CBFS, and mRmR. We compared the results by implementing two linear (SVM and LDA) and two non-linear (NB and MLP) machine learning classification algorithms. We also implemented the IDBN with simple criteria to avoid overfitting. The results show that the proposed pipeline and the IDBN perform slightly better than the linear and non-linear models implemented in this study. In the future, we would implement the proposed pipeline on SNP and DNA Methylation dataset to evaluate the model’s generalization.

## Data Availability

The original contributions presented in the study are included in the article/Supplementary Material, further inquiries can be directed to the corresponding authors.
